# The relationship between thyroid hormones sensitivity and hyperhomocysteinemia: a cross-sectional study based on Chinese health check-up population

**DOI:** 10.3389/fendo.2025.1634589

**Published:** 2025-10-02

**Authors:** Rui Gong, Jiao Xie, Lixia Yu, Yan Ling, Shi Wang, Rui Min, Sanping Xu

**Affiliations:** ^1^ Health Management Center, Union Hospital, Tongji Medical College, Huazhong University of Science and Technology, Wuhan, China; ^2^ Department of Breast and Thyroid Surgery, Union Hospital, Tongji Medical College, Huazhong University of Science and Technology, Wuhan, China; ^3^ School of Public health, Tongji Medical College, Huazhong University of Science and Technology, Wuhan, China

**Keywords:** thyroid hormone sensitivity, homocysteine, hyperhomocysteinemia, health check-up population, cardiovascular disease risk

## Abstract

**Background:**

It is unclear if impaired thyroid hormone sensitivity interacts with homocysteine (Hcy), a known risk factor for cardiovascular diseases (CVD), despite the fact that it has been identified as a prevalent metabolic condition.We aimed to analyze the association between thyroid hormone sensitivity indices and homocysteine levels in a Chinese health check-up population.

**Methods:**

Thyroid hormone sensitivity was assessed by the Thyroid Feedback Quantization Index (TFQI), the parameter TFQI (PTFQI), the TSH index (TSHI) and the thyrotropin thyroxine resistance index (TT4RI) and FT3/FT4 ratio. Linear regression analyses, logistic regression analyses and restricted cubic spline were used to investigate the relationship between thyroid hormone sensitivity and Hcy levels.

**Results:**

This study included 11144 medical examiners. Subjects with impaired thyroid hormone sensitivity had higher Hcy levels, according to the results (P < 0.001). Quartiles of TFQI, PTFQI, TSHI, and TT4RI were linked to Hcy levels, according to logistic regression analysis, and these associations persisted even after controlling for a number of risk factors. The odds ratio (95% CI) for Hyperhomocysteinemia (HHcy) in the highest quartile of TFQI was 1.294(1.114,1.504), for PTFQI was 1.293 (1.113, 1.503), and for TSHI was 1.222 (1.050, 1.422) (P < 0.001) after controlling for age, sex, body mass index, dyslipidemia, diabetes, and hypertension.

**Conclusions:**

A significant association between impaired thyroid hormone sensitivity and elevated Hcy levels exists in a Chinese population with normal thyroid function.

## 1 Introduction

Homocysteine (Hcy) is a sulfur-containing amino acid structurally similar to cysteine and is an intermediate in methionine metabolism. Hyperhomocysteinemia (HHcy) is characterized by pathologically elevated levels of Hcy in the blood, which result from disruptions in metabolic reactions involving essential vitamins (B6, B12, and folate) and enzymes, particularly methylenetetrahydrofolate reductase (MTHFR) (1). The prevalence of HHcy varies by region. The highest prevalence is found in Iran (73.1%) (2), followed closely by parts of Africa (62.3%) (3). In China, the prevalence of HHcy has increased from 27.5% to 37.2% over the past two decades (4), which is higher than that in the United States (6.9%) (5) and Canada (19.1%) (6). Nutritional deficiencies, alcohol consumption, untreated celiac disease, and prolonged use of proton pump inhibitors can all lead to elevated Hcy levels because proton pump inhibitors play a key role in Hcy metabolism (7–9). Elevated Hcy is also associated with atherosclerosis (10), chronic kidney disease (11), Parkinson’s disease (12), metabolic-associated steatohepatopathy (MASLD) (13), and thyroid disease (14, 15), among other diseases.

Thyroid hormones are biologically active substances synthesized and secreted by the thyroid gland, mainly including thyroxine (T4) and triiodothyronine (T3). These hormones influence cellular functions and metabolism primarily by binding to nuclear thyroid hormone receptors, which in turn regulate gene transcription (16). Previous studies have shown a negative correlation between thyroid hormone levels and Hcy levels. Patients with hyperthyroidism usually have lower Hcy levels compared to euthyroid individuals, whereas patients with hypothyroidism exhibit elevated Hcy levels (15, 17). However, some studies show that Hcy levels are significantly elevated in patients with hyperthyroidism (18), Therefore, more studies are needed to explore the relationship between thyroid hormone levels and Hcy levels. Further studies have revealed that mild acquired resistance to thyroid hormone may occur within the general population. the Thyroid Feedback Quartile Index (TFQI) has been identified as a new indicator of central sensitivity to thyroid hormone, and other composite indices include the Thyrotropin Resistance Index (TT4RI), the TSH Index (TSHI), and the Parametric TFQI (PTFQI). Additionally, peripheral variability of thyroid hormones can be assessed by the Free Thyroxine (FT3)/FT4 ratio (19). Thyroid hormone sensitivity has been linked to several disorders, such as hyperuricemia (20), elevated residual cholesterol levels (21), and vitamin D deficiency (22). However, there have been limited studies on the correlation between thyroid hormone sensitivity and Hcy levels in subjects with normal thyroid function. Therefore, in this study, we aimed to gain insight into the interactions and relationships between thyroid hormone sensitivity and Hcy levels in a Chinese population with normal thyroid function.

## 2 Materials and methods

### 2.1 Study population

This study enrolled participants (aged ≥ 18 years) who underwent a routine physical examination at the Health Management Center of Wuhan Union Hospital from January 2020 to December 2023. Participants with abnormal thyroid function, a history of thyroid disease, hormone replacement therapy, use of medications known to affect serum Hcy levels (determined by reviewing their past medical and medication history), with severe organ failure, or pregnancy were excluded. Ultimately, 11,144 subjects were recruited for the final analysis. The research was approved by the Ethics Committee of Union Hospital, Tongji Medical College, Huazhong University of Science and Technology. The study procedures adhered to the requirements of the Declaration of Helsinki. The requirement for written informed consent was waived due to the retrospective nature of the study.

### 2.2 Data collection

Physiologic parameters were collected: systolic blood pressure (SBP), diastolic blood pressure (DBP), weight and height. Laboratory parameters: total bilirubin (TBIL), direct bilirubin (DBIL), alanine aminotransferase (ALT), aspartate aminotransferase (AST), gamma-glutamyl transferase (GGT), triglycerides (TG), high-density lipoprotein cholesterol (HDL-C), low-density lipoprotein (LDL), total cholesterol (TC), blood urea nitrogen (BUN), creatinine (CR), urea nitrogen, uric acid (UA), fasting blood glucose (FBG), glycosylated hemoglobin (HbA1c), Hcy, FT4, FT4 and thyroid stimulating hormone (TSH). Dyslipidemia was defined as TG ≥ 1.7 mmol/L, HDL-C < 1.0 mmol/L, LDL-C ≥ 3.4 mmol/L, and C ≥ 5.2 mmol/L. Diabetes was defined as a fasting FBG≥7.0 mmol/L, use of diabetic medications, or a history of self-reported diabetes. Hypertension was defined as use of antihypertensive medications, sSBP ≥140 mmHg, or DBP ≥90 mmHg.

### 2.3 Indices of thyroid hormone sensitivity

Four different indices were calculated to assess central sensitivity to thyroid hormones as follows:

TFQI = (cdf)FT4 - (1 - cdf TSH);
TSHI = lnTSH (μIU/ml) + 0.1345 × FT4 (pmol/L);
TT4RI = FT4 (pmol/L) × TSH (μIU/ml);
PTHQI = Φ ((FT4 - μFT4)/σFT4) - (1 - Φ ((ln TSH - μln TSH)/σln TSH)).

Among them, TFQI was calculated using the cumulative distribution function and PTHQI was calculated using the standard normal cumulative distribution function, these indices measure how appropriately the TSH response aligns with circulating FT4. Higher values indicate lower central sensitivity to thyroid hormone. The peripheral index of thyroid hormone sensitivity is calculated as follows: FT3/FT4 ratio = FT3 (pmol/L)/FT4 (pmol/L).The higher the FT3/FT4 ratio, the better the peripheral sensitivity to thyroid hormone.

### 2.4 Statistic analysis

All analyses were performed using SPSS 26.0 (Chicago, IL, USA). Normality was examined by the Kolmogorov–Smirnov test. Skewed-distribution variables were described as median and interquartile range. Data of categorical variables were expressed as numbers (%) and the Chi-square test was used to compare groups. The difference between two groups was compared by the Mann-Whitney U test. Furthermore, multiple comparisons between groups were conducted using a general linear model with one-way analysis of covariance (ANCOVA) followed by Bonferroni *post hoc* tests, considering age and sex as covariates. P for trend was calculated by linear regression analyses. Multivariable linear regression analyses, adjusted for age, sex, BMI, diabetes, dyslipidemia, and hypertension, were used to examine the correlation between Hcy and thyroid parameters. Lastly, using three models that adjusted for potential confounding variables, stepwise multivariable logistic regression analysis was used to assess the relationships between HHcy and markers of thyroid hormone sensitivity. In Model1, we incorporated the basic demographic indicators of age and gender into the regression analysis as control variables; In Model 2, we further took into account the BMI (the most common indicator for weight) as control variables and continued to incorporate the regression analysis based on Model1; Finally, we added the three common metabolic-related chronic disease, diabetes, hyperlipidemia and hypertension as control variables in the Model 3.The adjusted dose–response relationships between thyroid hormone sensitivity indices and Hcy was presented using a restricted cubic spline function using 3 knots at the 10th, 50th and 90th percentiles (https://www.medsta.cn/). P < 0.05 (two-tailed) was considered statistically significant.

## 3 Results

### 3.1 Basic characteristics of the study population

The baseline characteristics of the study population are shown in **Table 1**. The HHcy population comprised 15.6% of the total. Male participants outnumbered female participants (P < 0.001). In the HHcy group, age, BMI, and the levels of BUN, CR, and UA were significantly higher compared to those in the non-HHcy group. The HHcy group also had a greater proportion of hypertension and dyslipidemia (P < 0.001). Notably, TFQI and PTFQI levels were significantly higher in the Hhcy group than in the non-HHcy group (P < 0.05). However, there were no significant differences in TSH, TSHI, TT4RI, FT3/FT4 ratio, FBG, HbA1c levels, or the proportion of diabetic patients.

**Table 1:** Comparison of basic characteristics between participants with and without HHcy.

Variables	Total n=11144	Non-HHcy n=9409	HHcy n=1735	*P* value
Male, n (%)	6946 (62.3)	5370 (57.1)	1576 (90.8)	< 0.001
Age, years	51 (44, 59)	51 (44, 58)	53 (45, 62)	< 0.001
SBP, mmHg	120 (110, 133)	120 (108, 132)	126 (114, 140)	< 0.001
DBP, mmHg	76 (68,84)	75 (68,83)	78 (70,86)	< 0.001
Dyslipidemia, n (%)	2265 (20.3)	1736 (18.5)	529 (30.5)	0.001
Hypertension, n (%)	600 (5.4)	491 (5.2)	109 (6.30)	< 0.001
Diabetes, n (%)	4536 (40.7)	3896 (41.4)	640 (36.90)	0.071
BMI, kg/m^2^	24.1 (22, 26.396)	24.08 (21.9, 26.3)	24.4 (22.4, 26.7)	< 0.001
FT3, pmol/L	13 (12.2, 13.9)	12.9 (12.2, 13.8)	13.2 (12.4, 14.1)	< 0.001
FT4, pmol/L	4.5 (4.2, 4.9)	4.5 (4.2, 4.9)	4.6 (4.3, 4.9)	< 0.001
TSH	1.726 (1.252, 2.399)	1.732 (1.261, 2.404)	1.695 (1.221,2.367)	0.1
TSHI	2.308 (1.974, 2.642)	2.31 (1.973, 2.642)	2.296 (1.978, 2.647)	0.763
TT4RI	22.47 (16.31, 31.23)	22.51 (16.33, 31.27)	22.20 (16.25, 30.92)	0.577
TFQI	0.002 (-0.27, 0.27)	-0.003 (-0.28, 0.26)	0.029 (-0.25, 0.30)	0.003
PTFQI	0.00 (-0.28, 0.263)	-0.003 (-0.286, 0.257)	0.026 (-0.262, 0.294)	0.003
FT3/FT4 ratio	0.349 (0.318, 0.38)	0.349 (0.319, 0.38)	0.349 (0.318, 0.383)	0.768
TG, mmol/L	1.32 (0.92, 2.00)	1.3 (0.9, 1.96)	1.46 (1.03, 2.23)	< 0.001
TC, mmol/L	4.93 (4.32, 5.6)	4.94 (4.35, 5.61)	4.81 (4.18, 5.53)	< 0.001
HDL_C, mmol/L	1.3 (1.09, 1.58)	1.33 (1.11, 1.6)	1.21 (1.02, 1.44)	< 0.001
LDL_C, mmol/L	2.895 (2.39, 3.44)	2.9 (2.4, 3.45)	2.85 (2.33, 3.42)	0.012
TBIL, μmmol/L	13.6 (11, 17.3)	13.5 (10.9, 17.1)	14.2 (11.2, 18.3)	< 0.001
DBIL, μmmol/L	4.3 (3.3, 5.6)	4.2 (3.3, 5.5)	4.6 (3.5, 6.0)	< 0.001
ALT, U/L	22 (16, 33)	22 (16, 33)	24 (17, 36)	< 0.001
AST, U/L	23 (19, 28)	23 (19, 28)	24 (20, 29)	< 0.001
GGT, U/L	22 (15, 35)	21 (14, 33)	26 (18, 42)	< 0.001
BUN, mg/dL	4.84 (4.07, 5.73)	4.8 (4.04, 5.68)	5.05 (4.23, 5.97)	< 0.001
CR, μmol/L	70.1 (59.2, 80.4)	67.8 (57.8, 78.3)	79.9 (72, 88.8)	< 0.001
UA, μmol/L	343.4 (278.3, 413.4)	334.1 (271.5, 402.8)	391.1 (329.4, 457.1)	0.003
FBG, mmol/L	4.92 (4.59, 5.38)	4.92 (4.6, 5.36)	4.9 (4.53, 5.41)	0.21
HbA1c, %	5.5 (5.2, 5.8)	5.5 (5.2, 5.7)	5.5 (5.2, 5.8)	0.861

Data are expressed mean ± SD or median (interquartile range) or number (%). Bold indicates P value < 0.05.HHcy, hyperhomocy steinemia; SBP, systolic blood pressure; DBP, diastolic blood pressure; BMI, body mass index; FT3, free triiodothyronine; FT4, free thyroxine;TSH, thyroid stimulating hormone; TSHI, TSH index; TT4RI, thyrotropin thyroxine resistance index, TFQI, thyroid feedback quantile-based index; PTFQI, Parametric thyroid feedback quantile-based index; TG, triglycerides; TC, total cholesterol; HDL-C, high-density lipoprotein cholesterol; LDL-C, low-density lipoprotein cholesterol; TBIL, total bilirubin; DBIL, direct bilirubin; ALT, alanine aminotransferase; AST, aspartate aminotransferase; GGT, gamma-glutamyl transferase; BUN, blood urea nitrogen; CR, creatinine; UA, uric acid; FBG, fasting blood glucose; HbA1c, glycated hemoglobin.

### 3.2 Correlation of thyroid hormone sensitivity with Hcy levels

**Figure 1:**
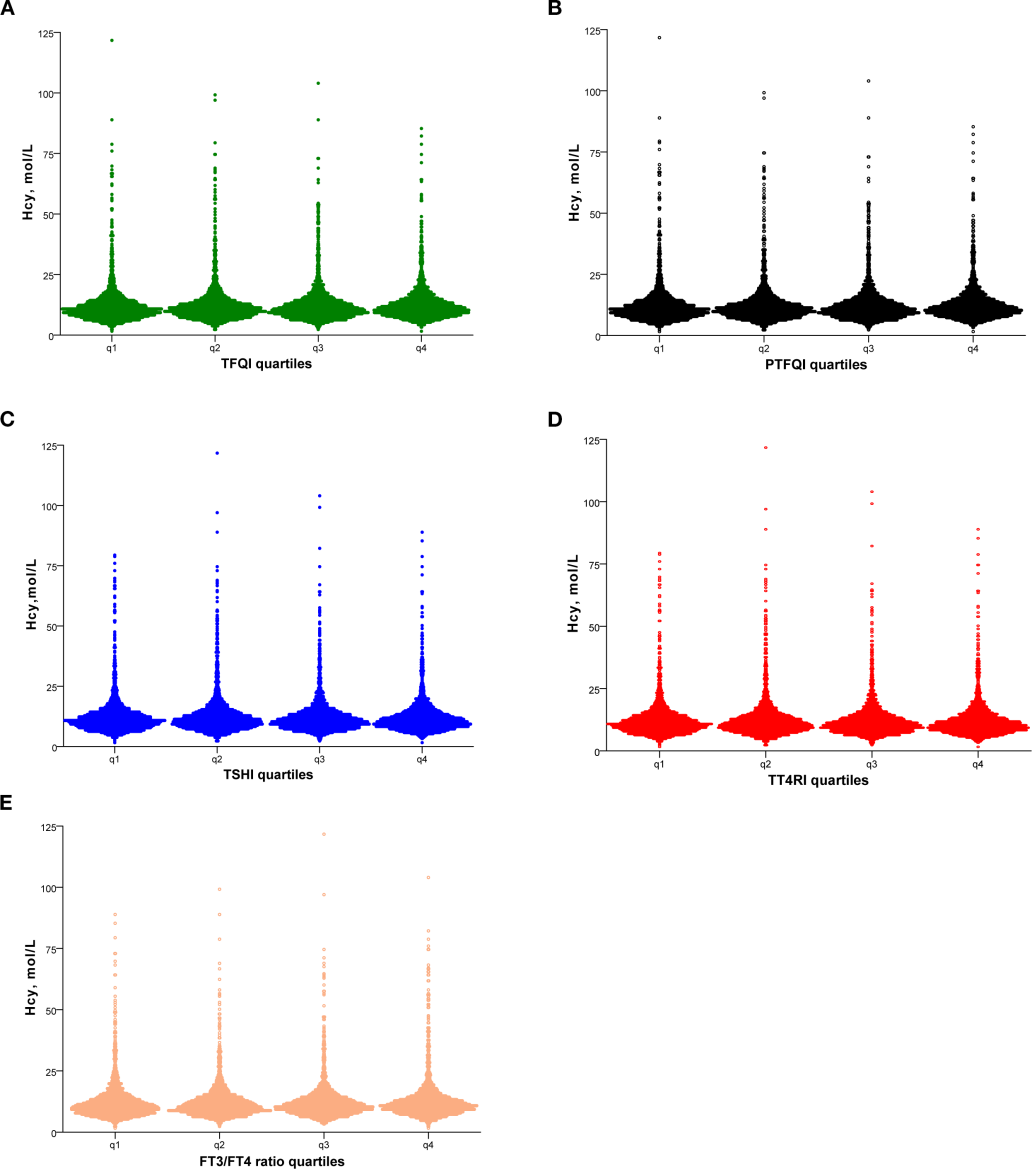
Hcy levels according to the quartiles of TFQI **(A)**, PTFQI **(B)**, TSHI **(C)**, TT4RI **(D)** and FT3/FT4 ratio **(E)**. Hcy, homocysteine; TFQI, thyroid feedback quantile-based index; PTFQI, parametric TFQI; TSHI, TSH index; TT4RI, thyrotropin thyroxine resistance index, FT3, free triiodothyronine; FT4, free thyroxine.

**Figure 1** showed the Hcy levels by quartile of indices of thyroid hormones sensitivity. Hcy levels increased linearly across TFQI, PTFQI, TSHI, and TT4RI (P < 0.05). After further adjustment for age and sex, Hcy concentrations progressively increased with higher quartiles of TFQI, PTFQI, TSHI, and TT4RI (**Table 2**). Multivariate linear regression analysis revealed that Hcy levels were positively associated with TFQI (β = 0.075, P < 0.001) and TT4RI (β = 0.074, P = 0.005), but not with FT3/FT4 ratio, TSHI, and PTFQI (**Table 3**).

**Table 2:** Hcy levels across the quartiles of TFQI, PTFQI, TSHI, TT4RI and FT3/FT4 ratio.

Variables	Q1	Q2	Q3	Q4	P value
TFQI	2.364(2.351,2.377)	2.390(2.377,2.403) ^**^	2.402(2.389,2.415) ^***^	2.430(2.417,2.443) ^***/###/++^	<0.001
PTFQI	2.364(2.351,2.377)	2.389(2.376,2.403) ^**/+++^	2.403(2.390,20416) ^***^	2.429(2.416,2.443) ^***/###/++^	<0.001
TSHI	2.374(2.360,2.387)	2.400(2.387,2.413) ^**^	2.397(2.383,2.410) ^*^	2.416(2.403,2.429) ^***/++^	<0.001
TT4RI	2.381(2.367,2.394)	2.396(2.383,2.410)	2.398(2.384,2.411)	2.412 (2.398,2.425) ^**^	0.016

Data are expressed as adjusted mean and 95% CI. P values were performed by analysis of covariance (ANCOVA) adjusted for age and gender. Hcy levels were transformed as LnHcy for analysis. Hcy, homocysteine; PTFQI, parametric TFQI; TFQI, thyroid feedback quantile-based index; TSHI, TSH index; TT4RI, thyrotropin thyroxine resistance index. Bold indicates ^*/#/+^ P < 0.05, ^**/##/++^P < 0.01, ^***/###/+++^P < 0.001; *Compared with Quartile 1; # Compared with Quartile 2; + Compared with Quartile 3.The quartiles of Hcy Levels were excluded from the table as not pass the test for Homogeneity of variance test. The results of the non-parameter test showed that there was not significant different among the quartiles of Hcy Levels(P = 0.215). TFQI, thyroid feedback quantile-based index; PTFQI, parametric TFQI; TSHI, TSH index; TT4RI, thyrotropin thyroxine resistance index.

**Table 3:** Linear regression analysis for the correlation of thyroid parameters and Hcy in all participants.

Variables	β (95% CI)	P value
TFQI	0.075 (0.052, 0.107)	<0.001
TT4RI	0.074(0.001, 0.005)	0.005

Adjusted R2 = 0.253. Durbin-Waston test=1.961. Dependent variable: Hcy.Independent variables selected method: Stepwise. Data are expressed as standardized coefficients (β) and 95% CI for B. P values were adjusted for age, gender, dyslipidemia, and hypertension. Independents FT3/FT4 ratio, PTFQI, TSHI were removed as P>0.05. Continuous variables with skewed distributions were Log(e)^ transformed for analysis, such as, Hcy, TT4RI and TSHI. TFQI, t thyroid feedback quantile-based index; TT4RI, thyrotropin thyroxine resistance index.

### 3.3 Association of sensitivity to thyroid hormones and elevated Hcy levels

**Figure 2:**
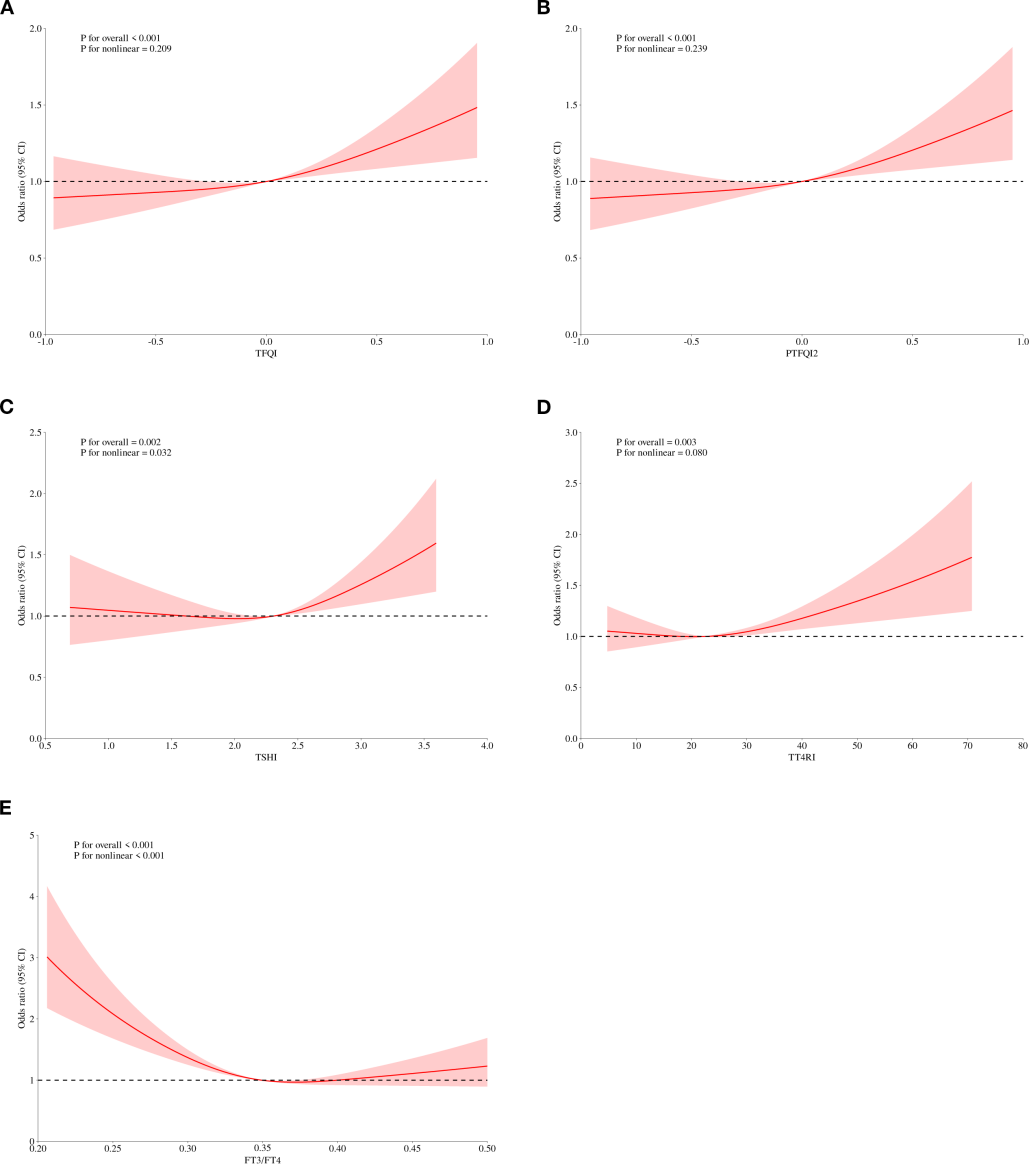
Adjusted dose–response relationship between thyroid hormones sensitivity indices and level of Hcy using the restricted cubic spline method. TFQI, thyroid feedback quantile-based index; PTFQI, parametric thyroid feedback quantile-based index; TT4RI, thyrotrophic thyroxine resistance index; TSHI, thyroid-stimulating hormone index.

We performed a logistic regression analysis on all patients to explore the potential relationship between sensitivity to thyroid hormones and elevated Hcy levels (**Table 4**). After adjusting for age, sex, BMI, diabetes, dyslipidemia, and hypertension, the odds ratio (95% CI) for having HHcy was as follows: TFQI in the highest quartile was 1.294 (1.114, 1.504), PTFQI was 1.293 (1.113, 1.503), and TSHI was 1.229 (1.056, 1.432) (P < 0.05). The full-size table is shown in the **Supplementary Material** (**Supplementary Table S1**). To further investigate, a restricted cubic spline analysis was conducted to assess the potential nonlinear association between thyroid hormone sensitivity and Hcy levels. After adjusting for sex, age, BMI, hypertension, dyslipidemia, and diabetes mellitus, a statistically significant nonlinear association was observed between TSHI and Hcy levels (**Figure 2**). Additionally, we conducted a subgroup analysis by sex, which also demonstrated a significant correlation between thyroid hormone sensitivity and HHcy (**Table 5**).

**Table 4:** Logistic regression analysis for the association of thyroid hormones sensitivity and elevated Hcy levels.

Variables	OR (95%C.I.)				P value
	Q1	Q2	Q3	Q4	
TFQI	-1.00~-0.273	-0.273~0.002	0.002~0.270	0.270~1	
Model 1	1.00(Ref)	1.065(0.914,1.240)	1.133(0.972,1.320)	1.288(1.109,1.496)	0.007
Model 2	1.00(Ref)	1.065(0.914,1.241)	1.134(0.973,1.321)	1.289(1.110,1.497)	0.007
Model 3	1.00(Ref)	1.066(0.914,1.243)	1.142(0.980,1.331)	1.294(1.114,1.504)	0.006
PTFQI	-1.00~-0.280	-0.280~0	0~0.263	0.263~1	
Model 1	1.00(Ref)	1.083(0.930,1.262)	1.145(0.983,1.334)	1.286 (1.107,1.493)	0.009
Model 2	1.00(Ref)	1.084(0.930,1.263)	1.146(0.984,1.335)	1.287 (1.108,1.495)	0.009
Model 3	1.00(Ref)	1.089(0.983,1.269)	1.155(0.991,1.346)	1.293 (1.113,1.503)	0.008
TSHI	~1.974	1.974~2.308	2.308~2.642	2.642~	
Model 1	1.00(Ref)	1.150(0.991,1.333)	1.050(0.902,1.223)	1.223(1.051,1.423)	0.042
Model 2	1.00(Ref)	1.148(0.990,1.332)	1.049(0.901,1.222)	1.222(1.050,1.422)	0.043
Model 3	1.00(Ref)	1.158(0.997,1.344)	1.064(0.913,1.240)	1.229(1.056,1.432)	0.040
TT4RI	~16.313	16.313~22.470	22.470~31.236	31.236~	
Model 1	1.00(Ref)	1.056(0.911,1.225)	1.059(0.911,1.231)	1.158(0.995,1.348)	0.297
Model 2	1.00(Ref)	1.056(0.911,1.225)	1.058(0.910,1.230)	1.158(0.995,1.347)	0.301
Model 3	1.00(Ref)	1.064(0.917,1.235)	1.074(0.923,1.249)	1.163(0.999,1.355)	0.284

Independent variables used Enter method. Data are expressed as odds ratio (OR) and 95% CI.

Model 1: adjusted for age and gender.

Model 2: adjusted for age, gender, and BMI.

Model 3: adjusted for age, gender, BMI, diabetes, dyslipidemia, and hypertension.

TFQI, thyroid feedback quantile-based index; PTFQI, parametric TFQI; TSHI, TSH index; TT4RI, thyrotropin thyroxine resistance index.

**Table 5:** Subgroup analyses stratified by gender.

Gender	TFQI,OR (95% CI)	PTFQI,OR (95% CI)	TSHI,OR (95% CI)	TyG,OR (95% CI)	TT4RI,OR (95% CI)
Male	1.25 (1.09 ~ 1.45) **	1.25 (1.08 ~ 1.44) **	1.16 (1.03 ~ 1.30) *	0.56 (0.46 ~ 0.68) ***	1.01 (1.00 ~ 1.01) **
Female	2.12 (1.39 ~ 3.23) ***	2.14 (1.40 ~ 3.27) ***	1.68 (1.20 ~ 2.36) **	1.36 (0.75 ~ 2.44)	1.02 (1.01 ~ 1.03) **

^*^P < 0.05, ^**^P< 0.01, ^***^P < 0.001; TFQI, thyroid feedback quantile-based index; PTFQI, parametric TFQI;TSHI, TSH index; TT4RI, thyrotropin thyroxine resistance index.

## 4 Discussion

Research indicates that Hcy plays a crucial role in cell signaling. It also affects cell proliferation, apoptosis, and inflammatory responses. Additionally, hydrogen sulfide, a metabolite derived from Hcy, is believed to have a protective effect on the health and function of blood vessels (23). HHcy is a metabolic disorder characterized by elevated levels of Hcy in the blood. Its association with cardiovascular disease (CVD) has attracted much attention. Numerous studies have confirmed that elevated plasma Hcy is an independent risk factor for CVD (24). Studies have shown that Hcy can accelerate atherogenesis through several mechanisms. These include oxidative stress, impaired endothelial function, inflammation, epigenetic changes, and disrupted lipoprotein metabolism (25, 26). In addition, HHcy is linked to several conditions, including erectile dysfunction (27), chronic kidney disease (28), and MASLD (13). Nonetheless, controversies regarding its assessment and management persist, primarily because of insufficient understanding of its clinical manifestations and distinct biochemical characteristics, which contribute to diagnostic delays in disorders related to homocysteine metabolism. Based on this, we conducted this study.

In this study, we observed that higher FT3 and FT4 levels were associated with HHcy. Thyroid hormones may affect Hcy metabolism through several potential mechanisms. First, thyroid hormones play a role in the regulation of enzymes related to Hcy metabolism. For instance, thyroid hormones regulate MTHFR expression and enhance its activity, which aids in remethylation of Hcy to methionine, thereby lowering plasma Hcy levels (29). Animal studies have shown that thyroidectomized rats have reduced MTHFR levels and activity, which suggests that hypothyroidism affects Hcy metabolism (30). Studies show that the kidney contains numerous enzymes involved in Hcy metabolism, such as betaine-homocysteine S-methyltransferase and MTHFR (31, 32). Other studies have confirmed that patients with hypothyroidism develop elevated Cr levels, which may reflect a reduced glomerular filtration rate. This suggests that thyroid hormone levels can affect Hcy levels by influencing the renal clearance of Hcy (33). Previous studies explored the relationship between thyroid function and Hcy levels, but no consistent conclusions have been reached. In fact, Zhou et al. reported in a meta-analysis that plasma Hcy concentrations were significantly higher in hypothyroid patients than in healthy controls and patients with subclinical hypothyroidism (SCH). Furthermore, hypothyroid patients showed a significant decrease in Hcy levels after levothyroxine treatment (34). Another study focused on the relationship between SCH patients and Hcy levels and demonstrated that Hcy levels were significantly higher in SCH patients than in healthy controls (35). However, another study showed that patients with hyperthyroidism had elevated Hcy levels. They also exhibited increased levels of methylmalonic acid, a marker of vitamin B12 deficiency. Based on these findings, the researchers hypothesized that elevated Hcy caused by hyperthyroidism may contribute to a functional vitamin B12 deficiency (18). These conflicting results were difficult to explain based on the metabolic mechanisms involved in clinical thyroid disorders. Therefore, Laclaustra et al. introduced new indices to assess thyroid hormone sensitivity more accurately (19), but the relationship within the euthyroid range, as assessed by sensitivity indices, remains unexplored.

Our study focused on the relationship between peripheral thyroid hormone sensitivity and Hcy levels in individuals with euthyroid status. There were significantly more men than women with HHcy. Age, BMI, the prevalence of hypertension and dyslipidemia, and levels of BUN, CR, and UA were significantly higher in the HHcy group compared to the non-HHcy group. TFQI and PTFQI levels were significantly higher in the HHcy group than in the non-HHcy group. After adjusting for age, sex, BMI, diabetes, dyslipidemia, and hypertension, we found that thyroid hormone sensitivity remains significantly associated with HHcy, but to a lesser extent. This result was consistent with previous findings (36), however, there is a certain difference between our linear analysis results and regression analysis, which may be due to the difference between linear (continuous) modeling and logical (classification) modeling. Additionally, subgroup analysis showed that thyroid hormone sensitivity was more strongly correlated with HHCY in women than in men. This difference may be attributed to hormonal variations, including the effect of estrogen on thyroid function and immune response (37, 38). However, not all thyroid sensitivity indicators were significantly correlated with Hcy levels in our study results. Similar findings have also been reported in other studies (39, 40), and differing results may be attributed to the formulas used to calculate each index. Both TFQI and PTFQI calculations include TSH and FT4 levels to assess thyroid hormone resistance. These indicators present a joint distribution of FT4 and TSH values. The advantage is that these indicators do not yield extreme thyroid hormone values. However, TT4RI and TSHI represent the extreme values of FT4 and TSH, respectively (41) and are relatively unstable, so the analysis results of each indicator are different. Overall, this retrospective analysis found that the risk of impaired thyroid hormone sensitivity and elevated Hcy levels is increased. Based on the above findings, thyroid dysfunction may influence Hcy levels by impacting B vitamin status. However, no studies have yet explored the mechanisms through which alterations in thyroid hormone sensitivity affect Hcy levels. This area warrants further in-depth investigation in the future.

However, our research also has some limitations. First, our study was a single-center retrospective study and did not examine the dynamic changes in thyroid hormone sensitivity indicators, which limited our ability to determine the causal relationship between thyroid hormone sensitivity and HHcy. Second, we lacked data on B vitamins, which may affect Hcy levels. Finally, we did not account for the confounding effects of lifestyle factors such as smoking, physical activity, and eating patterns. These limitations should be considered when interpreting our findings.

In conclusion, we found that impaired thyroid hormone sensitivity indices were associated with elevated Hcy levels in individuals with normal thyroid function. This study provides new insights into the interrelationship between thyroid hormones and Hcy metabolism and establishes a solid theoretical framework for future clinical interventions. Future research should further investigate the mechanisms by which thyroid hormones influence Hcy levels. Additionally, studies should explore the therapeutic potential of modulating thyroid hormone activity to prevent or treat HHcy.

## Data Availability

The raw data supporting the conclusions of this article will be made available by the authors, without undue reservation.
