# Supplementary material for: The relationship between thyroid hormones sensitivity and hyperhomocysteinemia: a cross-sectional study based on Chinese health check-up population
Source: Front Endocrinol (Lausanne). 2025 Oct 2;16:1634589. doi: 10.3389/fendo.2025.1634589 (PMC12527862; doi:10.3389/fendo.2025.1634589)
Supplement: Supplementary file 1 [file DataSheet1.docx]

Table S1. Logistic regression analysis for the association of thyroid hormones sensitivity and elevated Hcy levels（full size）

|  | **Model 1** | | | | **Model 2** | | | | **Model 3** | | | |
| --- | --- | --- | --- | --- | --- | --- | --- | --- | --- | --- | --- | --- |
|  | **OR** | ***P*-value** | **95% CI** | | **OR** | ***P*-value** | **95% CI** | | **OR** | ***P*-value** | **95% CI** | |
|  |  |  | **LOWERR** | **UPPER** |  |  | **LOWERR** | **UPPER** |  |  | **LOWERR** | **UPPER** |
| TFQI |  | 0.007 |  |  |  | 0.007 |  |  |  | 0.006 |  |  |
| TFQI(Q2) | 1.065 | 0.423 | 0.914 | 1.24 | 1.065 | 0.418 | 0.914 | 1.241 | 0.006 | 0.414 | 0.914 | 1.243 |
| TFQI(Q3) | 1.133 | 0.11 | 0.972 | 1.319 | 1.134 | 0.107 | 0.973 | 1.321 | 0.414 | 0.089 | 0.980 | 1.331 |
| TFQI(Q4) | 1.288 | **0.001** | 1.109 | 1.496 | 1.289 | **0.001** | 1.110 | 1.497 | 0.089 | **0.001** | 1.114 | 1.504 |
| GENDER | 0.137 | 0.000 | 0.116 | 0.162 | 0.138 | 0.000 | 0.117 | 0.164 | 0.001 | 0.000 | 0.116 | 0.163 |
| AGE | 1.02 | 0.000 | 1.015 | 1.025 | 1.020 | 0.000 | 1.015 | 1.025 | 0.000 | 0.000 | 1.011 | 1.021 |
| BMI |  |  |  |  | 1.010 | 0.250 | 0.993 | 1.026 | 0.000 | 0.369 | 0.991 | 1.024 |
| Hypertension |  |  |  |  |  |  |  |  | 0.369 | 0.000 | 1.381 | 1.771 |
| Diabetes |  |  |  |  |  |  |  |  | 0.000 | 0.031 | 0.627 | 0.978 |
| Dyslipidemia |  |  |  |  |  |  |  |  | 0.031 | 0.144 | 0.972 | 1.216 |
| Constant | 0.093 | 0.000 |  |  | 0.074 | 0.007 |  |  | 0.144 | 0.000 |  |  |
| Adjusted R^2^= | 0.138 |  |  |  | 0.138 |  |  |  | 0.146 |  |  |  |
| PTFQI(Ref. Q1 ) |  | 0.009 |  |  |  | 0.009 |  |  |  | 0.008 |  |  |
| PTFQI(Q2) | 1.083 | 0.306 | 0.930 | 1.262 | 1.084 | 0.302 | 0.930 | 1.263 | 1.089 | 0.277 | 0.934 | 1.269 |
| PTFQI(Q3) | 1.145 | 0.082 | 0.983 | 1.334 | 1.146 | 0.080 | 0.984 | 1.335 | 1.155 | 0.066 | 0.991 | 1.346 |
| PTFQI(Q4) | 1.286 | **0.001** | 1.107 | 1.493 | 1.287 | **0.001** | 1.108 | 1.495 | 1.293 | **0.001** | 1.113 | 1.503 |
| GENDER | 0.137 | 0.000 | 0.116 | 0.162 | 0.138 | 0.000 | 0.117 | 0.164 | 0.138 | 0.000 | 0.116 | 0.163 |
| AGE | 1.020 | 0.000 | 1.015 | 1.025 | 1.020 | 0.000 | 1.015 | 1.025 | 1.016 | 0.000 | 1.011 | 1.021 |
| BMI |  |  |  |  | 1.010 | 0.243 | 0.993 | 1.026 | 1.008 | 0.361 | 0.991 | 1.024 |
| Hypertension |  |  |  |  |  |  |  |  | 1.564 | 0.000 | 1.381 | 1.771 |
| Diabetes |  |  |  |  |  |  |  |  | 0.785 | 0.033 | 0.628 | 0.980 |
| Dyslipidemia |  |  |  |  |  |  |  |  | 1.087 | 0.143 | 0.972 | 1.217 |
| Constant | 0.093 | 0.000 |  |  | 0.073 | 0.000 |  |  | 0.082 | 0.000 |  |  |
| Adjusted R^2^= | 0.138 |  |  |  | 0.138 |  |  |  | 0.145 |  |  |  |
| TSHI(Ref. Q1 ) |  | 0.042 |  |  |  | 0.043 |  |  |  | 0.040 |  |  |
| TSHII(Q2) | 1.150 | 0.066 | 0.991 | 1.334 | 1.148 | 0.068 | 0.990 | 1.332 | 1.158 | 0.054 | 0.997 | 1.344 |
| TSHI(Q3) | 1.050 | 0.526 | 0.902 | 1.223 | 1.049 | 0.535 | 0.901 | 1.222 | 1.064 | 0.426 | 0.913 | 1.240 |
| TSHI(Q4) | 1.223 | **0.009** | 1.051 | 1.423 | 1.222 | **0.010** | 1.050 | 1.422 | 1.229 | **0.008** | 1.056 | 1.432 |
| GENDER | 0.136 | 0.000 | 0.115 | 0.161 | 0.137 | 0.000 | 0.116 | 0.162 | 0.137 | 0.000 | 0.115 | 0.162 |
| AGE | 1.020 | 0.000 | 1.015 | 1.025 | 1.020 | 0.000 | 1.015 | 1.025 | 1.016 | 0.000 | 1.011 | 1.021 |
| BMI |  |  |  |  | 1.009 | 0.270 | 0.993 | 1.026 | 1.007 | 0.395 | 0.991 | 1.024 |
| Hypertension |  |  |  |  |  |  |  |  | 1.565 | 0.000 | 1.383 | 1.772 |
| Diabetes |  |  |  |  |  |  |  |  | 0.794 | 0.041 | 0.636 | 0.991 |
| Dyslipidemia |  |  |  |  |  |  |  |  | 1.090 | 0.134 | 0.974 | 1.219 |
| Constant | 0.096 | 0.000 |  |  | 0.077 | 0.000 |  |  | 0.086 | 0.000 |  |  |
| Adjusted R^2^= | 0.137 |  |  |  | 0.137 |  |  |  | 0.145 |  |  |  |
| TT4RI(Ref. Q1 ) |  | 0.297 |  |  |  | 0.301 |  |  |  | 0.284 |  |  |
| TT4RI(Q2) | 1.056 | 0.468 | 0.911 | 1.225 | 1.056 | 0.470 | 0.911 | 1.225 | 1.064 | 0.412 | 0.917 | 1.235 |
| TT4RI(Q3) | 1.059 | 0.453 | 0.911 | 1.231 | 1.058 | 0.464 | 0.910 | 1.230 | 1.074 | 0.354 | 0.923 | 1.249 |
| TT4RI(Q4) | 1.158 | 0.057 | 0.995 | 1.348 | 1.158 | 0.059 | 0.995 | 1.347 | 1.163 | 0.052 | 0.999 | 1.355 |
| GENDER | 0.136 | 0.000 | 0.115 | 0.161 | 0.137 | 0.000 | 0.116 | 0.163 | 0.137 | 0.000 | 0.115 | 0.162 |
| AGE | 1.020 | 0.000 | 1.015 | 1.025 | 1.020 | 0.000 | 1.015 | 1.025 | 1.016 | 0.000 | 1.011 | 1.021 |
| BMI |  |  |  |  | 1.009 | 0.267 | 0.993 | 1.026 | 1.007 | 0.392 | 0.991 | 1.024 |
| Hypertension |  |  |  |  |  |  |  |  | 1.565 | 0.000 | 1.383 | 1.772 |
| Diabetes |  |  |  |  |  |  |  |  | 0.794 | 0.042 | 0.636 | 0.992 |
| Dyslipidemia |  |  |  |  |  |  |  |  | 1.089 | 0.138 | 0.973 | 1.218 |
| Constant | 0.099 | 0.000 |  |  | 0.079 | 0.000 |  |  | 0.089 | 0.000 |  |  |
| Adjusted R^2^= | 0.137 |  |  |  | 0.137 |  |  |  | 0.144 |  |  |  |

Dependent variable：HHcy. Independent variables used Enter method. Data are expressed as odds ratio (OR) and 95% CI.

Model 1: adjusted for age and gender.

Model 2: adjusted for age, gender, and BMI.

Model 3: adjusted for age, gender, BMI, diabetes, dyslipidemia, and hypertension.

TFQI, thyroid feedback quantile-based index; PTFQI, parametric TFQI;TSHI, TSH index; TT4RI, thyrotropin thyroxine resistance index.
